# LGR5-Positive Supporting Cells Survive Ototoxic Trauma in the Adult Mouse Cochlea

**DOI:** 10.3389/fnmol.2021.729625

**Published:** 2021-10-05

**Authors:** Natalia Smith-Cortinez, Rana Yadak, Ferry G. J. Hendriksen, Eefje Sanders, Dyan Ramekers, Robert J. Stokroos, Huib Versnel, Louise V. Straatman

**Affiliations:** ^1^Department of Otorhinolaryngology and Head & Neck Surgery, University Medical Center Utrecht, Utrecht, Netherlands; ^2^UMC Utrecht Brain Center, University Medical Center Utrecht, Utrecht University, Utrecht, Netherlands

**Keywords:** inner ear regeneration, deafness, LGR5+ supporting cells, ototoxicity, adult mammalian cochlea

## Abstract

Sensorineural hearing loss is mainly caused by irreversible damage to sensory hair cells (HCs). A subgroup of supporting cells (SCs) in the cochlea express leucine-rich repeat-containing G-protein coupled receptor 5 (LGR5), a marker for tissue-resident stem cells. LGR5+ SCs could be used as an endogenous source of stem cells for regeneration of HCs to treat hearing loss. Here, we report long-term presence of LGR5+ SCs in the mature adult cochlea and survival of LGR5+ SCs after severe ototoxic trauma characterized by partial loss of inner HCs and complete loss of outer HCs. Surviving LGR5+ SCs (confirmed by GFP expression) were located in the third row of Deiters’ cells. We observed a change in the intracellular localization of GFP, from the nucleus in normal-hearing to cytoplasm and membrane in deafened mice. These data suggests that the adult mammalian cochlea possesses properties essential for regeneration even after severe ototoxic trauma.

## Introduction

Hearing loss affects almost 500 million people worldwide, including 34 million children (World Health Organization, 2021), and it has been estimated that 900 million people could have disabling hearing loss by 2050 (Wilson et al., 2017; Chadha et al., 2018). Diverse etiologies, including aging, trauma, noise exposure, ototoxic drugs or genetic diseases, cause irreversible damage to sensory hair cells (HCs) in the cochlea (Edge and Chen, 2008; Mittal et al., 2017). While hearing aids and cochlear implants often result in recovery of hearing in hearing-impaired patients, a key problem is limited quality of the auditory percept (Caldwell et al., 2017; Lesica, 2018; Peters et al., 2018). Regeneration of cochlear HCs from endogenous cochlear stem cells could be a novel approach to improve hearing without the need of an electronic device.

In non-mammalian vertebrates, HC loss triggers spontaneous regeneration through re-entry of supporting cells (SCs) into cell cycle and transdifferentiation into new HCs (Dooling et al., 1997). In mammals, it has been described that a subset of SCs from the mouse and human cochlea have stem cell characteristics, and possess the potential to differentiate into new HCs *in vitro* and *in vivo* (Warchol et al., 1993; Li et al., 2003; White et al., 2006; McLean et al., 2017; Shu et al., 2019). The differentiation of SCs into HCs is mainly controlled by the Notch and Wnt signaling pathways which promote cell proliferation and differentiation (Chai et al., 2011, 2012; Mizutari et al., 2013; Li et al., 2015; Żak et al., 2015). The leucine-rich repeat-containing G-protein coupled receptor 5 (LGR5) is a membrane receptor in the Wnt pathway, which has been described as a stem-cell marker in different organs including the cochlea. It is expressed in a subgroup of SCs which give rise to HCs during murine embryonic development (Groves, 2010; Shi et al., 2012, 2013; Bramhall et al., 2014; Żak et al., 2016). Potentially these LGR5 positive (LGR5+) SCs can be utilized as endogenous stem cells for HC regeneration to treat hearing loss including deafness (severe hearing loss).

The differentiation into sensory HCs has been achieved by different experimental approaches using 3D-grown inner ear organoids derived from human pluripotent stem cells (Koehler et al., 2013), mouse embryonic stem cells (Koehler and Hashino, 2014) and human fetal cochlear progenitors (Roccio et al., 2018). Moreover, the differentiation of LGR5+ SCs into sensory HCs has also been observed by culturing 3D-grown cochlear organoids from the neonatal mouse cochlea after manipulation of the Wnt and/or Notch signaling pathways (Chai et al., 2012; Shi et al., 2012; McLean et al., 2017; Roccio and Edge, 2019). Preliminary results of one study demonstrated that myosin VIIA positive hair cell-like cells can even be regenerated from human adult inner ear epithelium *in vitro* (McLean et al., 2017).

Interestingly, neonatal LGR5+ SCs have been shown to survive and retain regeneration potential after an ototoxic trauma with neomycin *in vitro* (Zhang et al., 2017). Moreover, after selective ablation of HCs, LGR5+ SCs act as region-specific HC progenitors and are capable of both mitotic and non-mitotic HC regeneration in the neonatal mouse cochlea (Wang et al., 2015). Although it is known that LGR5+ SCs are still present in the organ of Corti in the adult mouse (Chai et al., 2011; Shi et al., 2012) their long-term presence in the mature mouse (after p60) has not been elucidated. Moreover, and critical toward therapeutic applications, it is unknown whether the LGR5+ SCs survive an ototoxic trauma in the adult cochlea. Therefore, we examined LGR5 expression in the organ of Corti 1 week after ototoxic medication in adult Lgr5^GFP^ mice.

Here, we report for the first time the survival of LGR5+ SCs in the deafened adult cochlea, using a mouse model of ototoxicity previously established in our lab (Jansen et al., 2013) in adult Lgr5^GFP^ transgenic mice. LGR5+ SCs might therefore be target cells for therapeutic treatment to regenerate HCs even in adulthood.

## Results

### Auditory Brainstem Responses and Cochlear Anatomical Organization Are Similar in Normal-Hearing Lgr5^GFP^ and Wild Type Adult Mice (p30 and p100)

**FIGURE 1:**
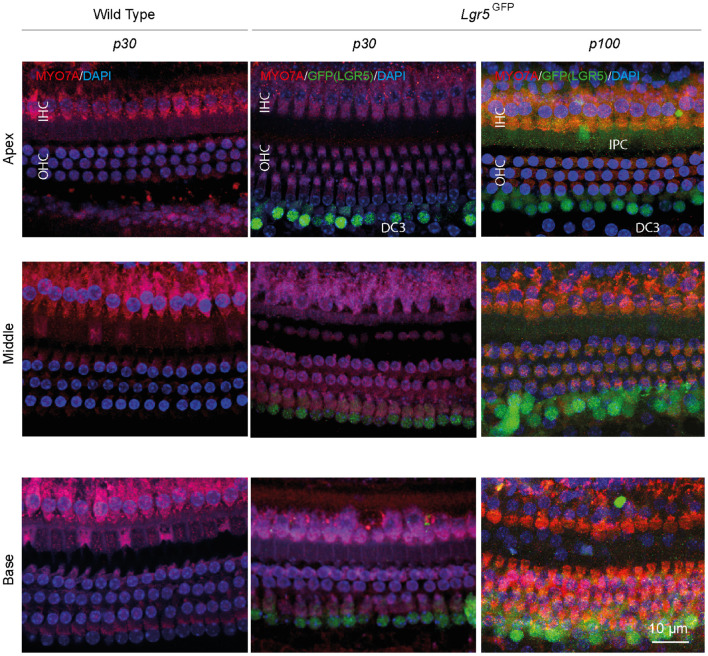
Anatomy of the organ of Corti in normal-hearing wild type (WT) and Lgr5^GFP^ adult mice. Representative images of immunofluorescence microscopy of apex, middle and base in whole mount dissections of the cochlea of WT and Lgr5^GFP^ adult (p30 and p100) mice stained with myosin VII A (MYO7A) in red, GFP (LGR5) in green and DAPI in blue. GFP (LGR5) was detected particularly in the 3rd row of Deiters’ cells (DC3s) and, to a lesser extent, in the inner pillar cells (IPCs). No changes in GFP (LGR5) expression were found between p30 and p100 mice. Bar = 10 μm. n_WT_ = 3; n_Lgr__5G__FP__(p__30__)_ = 7; n_Lgr__5G__FP(p__100__)_ = 3 (representative images of 1 cochlea per group).

To determine the hearing performance of the Lgr5^GFP^ transgenic adult mice relative to WT mice, we recorded click-evoked auditory brainstem responses (ABRs) in both groups. The p30 WT and Lgr5^GFP^ mice had similar ABR waveforms and their ABR thresholds were similar (difference smaller than 5 dB, data not shown). Immunofluorescence microscopy of whole-mount dissections of the cochlea of WT (p30) and Lgr5^GFP^ mice (p30 and p100) showed the typical image of one row of inner hair cells (IHCs) and three rows of outer hair cells (OHCs) in the apex, middle and base of the cochlea, expressed as MYO7A+ cells (in red, Figure 1). Moreover, we could clearly observe LGR5+ SCs in the apex, middle and base of the cochlea of all Lgr5^GFP^ adult mice, even until p100 (in green, Figure 1). The LGR5+ SCs were located in the third row of Deiters’ cells (DC3s) as well as, to a lesser extent, in the inner pillar cells (IPCs) in the cochlea of p30 and p100 Lgr5^GFP^ mice (Figure 1).

### Ototoxic Trauma Causes Severe Hearing Loss, Extensive Loss of Outer Hair Cells but Survival of LGR5+ SCs

**FIGURE 2:**
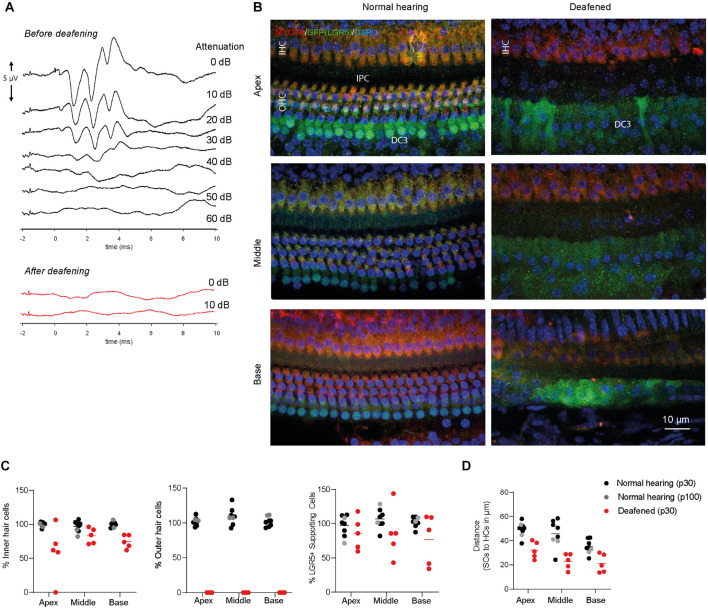
LGR5+ supporting cells are detected in the deafened Lgr5^GFP^ adult mice. **(A)** Representative auditory brain stem responses (ABRs) of one Lgr5^GFP^ mouse before (in black) and 1 week after (in red) deafening. **(B)** Representative images of immunofluorescence microscopy of apex, middle and base in whole-mount dissections of the cochlea of Lgr5^GFP^ normal-hearing and deafened mice stained with myosin VIIA (MYO7A) in red, GFP (LGR5) in green and DAPI in blue. MYO7A stainings showed that the outer hair cells (OHCs) were completely abolished after deafening, with partial preservation of inner hair cells (IHCs). Compared to the normal cochlea, the GFP (LGR5) expression after deafening was still present in the third row of Deiters’ cells (DC3s) and showed a cytoplasmic sublocalization and a more diffuse staining. **(C)** Cell counts, in percentage, for apex, middle and base of the cochlea of normal-hearing (p30 in black and p100 in gray) and deafened (p30) Lgr5^GFP^ mice. **(D)** Distance in μm from GFP + SCs to MYO7A+ IHCs. Bar = 10 μm. n_normal hearing(p__30__)_ = 6; n_normal hearing(p__100__)_ = 2 n_deafened(p__30__)_ = 5, mean.

Animals had normal thresholds before deafening (approximately 45 dB peak equivalent sound pressure level, peSPL), as observed in click-evoked ABRs (Figure 2A). One week after ototoxic trauma, mice showed little or no click-evoked ABR (Figure 2A), so the ABR thresholds were near the upper limitation of the recordings (>90 dB peSPL), confirming successful deafening after 7 days. Two animals with significant residual hearing (threshold shifts < 25 dB) were excluded from the analyses. Immunofluorescence microscopy of cochlear whole-mount dissections showed that the ototoxic medication destroyed all OHCs in the apex, middle and base (Figures 2B,C) and the expression of MYO7A (in red, Figure 2B) indicated an average survival of 60–80% of IHCs (Figures 2B,C). Interestingly, LGR5 (GFP) was still expressed in DC3s in the apex, middle and base of cochleas from deafened mice (in green, Figures 2B,C). However, IPCs seemed to have lost the LGR5 (GFP) expression (Figure 2B). Notably, some of the deafened cochleas showed two rows of LGR5+ SCs and these were located significantly closer to IHCs than in cochleas from normal-hearing mice [*p* < 0.001, *F*(1, 9) = 27; Figure 2D]. Furthermore, we observed no changes in the number of LGR5+ SCs located in DC3s after deafening [*p* = 0.17, *F*(1, 9) = 2.2; Figure 2C, right panel].

**FIGURE 3:**
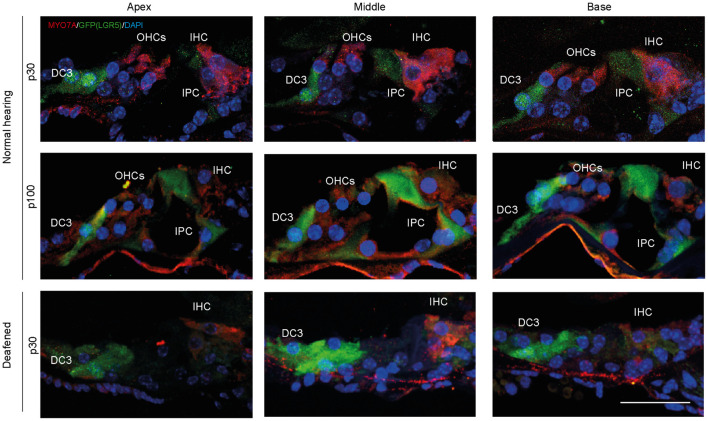
After deafening inner pillar cells (IPCs) lose LGR5 expression. Representative images of immunofluorescence microscopy of cryosections of the cochlea of normal-hearing (p30 and p100) and deafened Lgr5^GFP^ mice stained with myosin VII A (MYO7A) in red, GFP (LGR5) in green and DAPI in blue. IPCs of normal-hearing animals showed LGR5 expression, which disappeared after deafening. Bar = 10 μm. n_normal hearing(p__30__)_ = 6; n_normal hearing(p__100__)_ = 1 n_deafened(p__30__)_ = 5 (representative images of 1 cochlea per group).

Analysis of MYO7A and LGR5 (GFP) expression in cryosections showed that in control cochleas (up to p100) LGR5 (GFP) was present in DC3s and IPCs, and after deafening LGR5 (GFP) was present only in DC3s (Figure 3). Furthermore, MYO7A expression was observed mainly in IHCs in cochleas from deafened mice and in IHC and OHC in cochleas from normal-hearing mice (Figure 3).

### GFP Changes Its Subcellular Localization After Deafening

**FIGURE 4:**
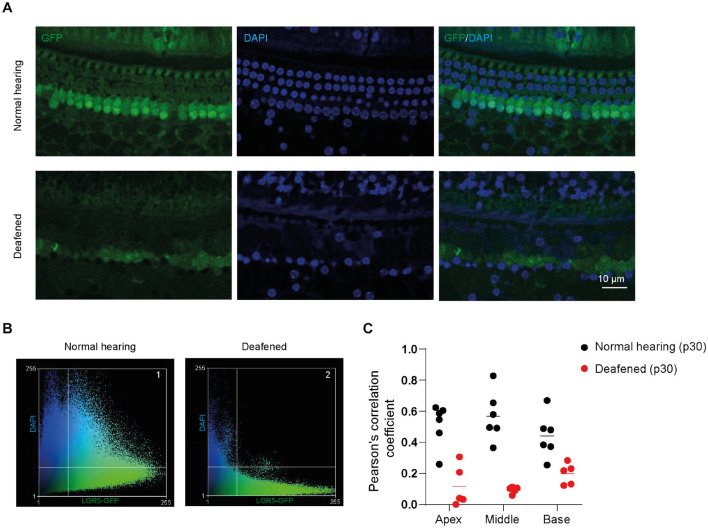
GFP expression is located in different subcellular compartments in the cochlea of normal-hearing and deafened mice. **(A)** Representative images of immunofluorescence microscopy of whole-mount dissections of the cochlea of normal-hearing and deafened Lgr5^GFP^ mice stained with GFP in green, DAPI in blue and merged images. **(B)** Scatter plot of green intensity vs. blue intensity of images in panel **(A)**. Square 1 represents the pixels that are expressed in both channels in normal hearing and square 2 represents the pixels that are expressed in both channels in deafened. **(C)** Pearson’s correlation coefficient (PCC) calculated for the apex, middle and base of the cochlea of normal-hearing and deafened Lgr5^GFP^ mice. Bar = 10 μm. n_normal hearing(p__30__)_ = 6; n_deafened(p__30__)_ = 5.

In the immunofluorescence data, we observed that GFP expression was mainly localized in the nuclei and cytoplasm of SCs in cochleas from normal-hearing mice and in the cytoplasm and plasma membrane (PM) of SCs in cochleas from deafened mice. To quantify the subcellular localization of GFP, we calculated Pearson’s correlation coefficient (PCC) in z-stacks taken for GFP (green) and DAPI (blue) in apex, middle and base of normal and deafened cochleas. We observed that GFP is present in the nuclei and to a lesser extent in the cytoplasm of SCs in control cochleas (Figure 4A, top panels) and mainly in the cytoplasm and PM of SCs in the deafened cochlea (Figure 4A, bottom panels). After plotting the green intensities vs. blue intensities of images in Figure 4A we can observe that in the normal conditions there is a gradient of pixels that co-localize in both channels (square 1, Figure 4B) whereas in deafened conditions there are pixels that either express DAPI or GFP but not both (square 2, Figure 4B). According to PCC, which is independent from fluorophore intensity, the co-localization was significantly lower in deafened mice (characterized by a low PCC in the apex, middle and base) than in normal-hearing mice (characterized by a high PCC in the apex, middle and base) [*F*(1, 9) = 39, *p* < 0.001; Figure 4C]. These results suggest that the subcellular localization of GFP is mainly nuclear in SCs of the normal-hearing mice and non-nuclear after deafening.

## Discussion

In this study the presence of LGR5+ SCs in cochleas of adult normal-hearing and deafened mice was evaluated *in vivo*. In normal-hearing adult Lgr5^GFP^ transgenic mice, with a hearing threshold similar to wild type (WT) littermates, LGR5+ SCs were found in the DC3s and, to a lesser extent, in the IPCs. One week after deafening, using a single dose of ototoxic co-medication of kanamycin and furosemide, there was survival of LGR5+ SCs in adult Lgr5^GFP^ transgenic mice, even though there was extensive loss of OHCs and substantial loss of IHCs. Interestingly, a change in subcellular localization of GFP in SCs was observed, which was expressed in the nuclei of SCs in normal cochleas and in the cytoplasm of SCs in deafened cochleas.

### Potential Endogenous Cochlear Stem Cells During Adulthood

Since the generation of the Lgr5^GFP^ transgenic mice (Barker et al., 2007) the LGR5 expression has been described in many tissues with known or previously unknown regeneration potential. In the neonatal mouse cochlea some SCs that express LGR5 have progenitor potential and can regenerate into new HCs *in vitro* and *in vivo* (Chai et al., 2012; Li et al., 2015; Ni et al., 2016; Chen et al., 2017). However, cochlear LGR5 expression has not been thoroughly described for adult mice, which is important since the majority of hearing-disabled people are adults (Cunningham and Tucci, 2017), so studying the role of LGR5+ SCs in adulthood is clinically very relevant. It has been shown that LGR5 expression gradually decreases until the second postnatal week and that it remains detectable only in the DC3s in the adult (p30) cochlea (Chai et al., 2011). Chai et al. (2011) also described that LGR5 expression is only detected in IPCs until p12. In contrast, another study showed that LGR5 is expressed in the adult (p30 and p60) mouse cochlea in the DC3s as well as in the IPCs (Shi et al., 2012). Our data further confirm these latter findings since we observed LGR5 expression even at p100 in DC3s and IPCs. As Shi et al., we performed immunofluorescence stainings using anti-GFP antibodies to increase the detection of LGR5, whereas Chai et al. (2011) did not use anti-GFP antibodies in their immunofluorescence, which potentially resulted in absence of a detectable LGR5 signal in the IPCs in mice older than p12. Moreover, in the present study no deterioration was found of LGR5 expression between p30 and p100. To our knowledge, this is the first study showing LGR5 expression in both DC3s and IPCs until p100 in Lgr5^GFP^ transgenic mice. This indicates that LGR5 expression in the cochlear SCs does not deteriorate during adulthood and suggests long-term availability of target cells for regenerative therapy for the adult cochlea.

### LGR5+ Cell Survival and Regenerative Capacities After Severe Ototoxic Trauma

Interestingly, in neonatal mouse cochlear tissue, it has been shown that after application of neomycin *in vitro* to induce HC loss, LGR5+ progenitors showed increased ability to proliferate and regenerate HCs 4 days after the ototoxic trauma (Zhang et al., 2017). For the adult mouse cochlea, this is the first study showing survival of LGR5+ SCs after deafening, which were located in the DC3s after deafening. This strongly suggests that the deafened mammalian cochlea retains regenerative potential, and hence therapeutic opportunities targeting the LGR5+ SCs could arise. A pioneering clinical trial has already shown that modulating the Wnt and Notch signaling pathway with a recently commercialized drug (FX322) in patients with sensorineural hearing loss improves speech recognition (both in quiet and in noise) 90 days after treatment (McLean et al., 2021). This report, including only patients with noise-induced or idiopathic sudden SNHL, supports the hypothesis that Wnt-responsive SCs with regeneration potential are present in human, even after deafness. The fact that we also found surviving LGR5+ progenitors after ototoxic trauma opens an opportunity for this treatment of ototoxically induced hearing loss as well.

The survival of SCs 1 week after an ototoxic event, even when resulting in extensive HC loss, is in accordance with previous studies and indicates that these are potentially less susceptible to ototoxic trauma. This is probably a result of less uptake of aminoglycoside (like kanamycin) in SCs, compared to HCs (Aran et al., 1995; Richardson et al., 1997, 1999; Young and Raphael, 2007; Taylor et al., 2012). Also in long-term experiments with mice deafened with 1 dose of kanamycin and bumetanide, survival of SCs 6 months after ototoxic trauma was shown, even when there was complete loss of IHCs and OHCs; however, it was not determined if the surviving SCs had progenitor potential (Taylor et al., 2012). Some other studies supported the hypothesis that there are SCs with regenerative capacities after ototoxic trauma: In adult guinea pigs treated with neomycin, the loss of HCs was accompanied by increasing number of dividing SCs 4 days after treatment, suggesting SCs were proliferating (Young and Raphael, 2007). Furthermore, in deafened adult guinea pigs, ototoxic trauma was accompanied by loss of IHCs and OHCs and survival of SCs. In these animals Atoh1 gene therapy improved regeneration and differentiation of new HCs 4 days (Izumikawa et al., 2005), 30 and 60 days (Kawamoto et al., 2003) after gene therapy.

In our study, we assessed expression of LGR5 1 week after deafening which represents an intermediate step between an acute and a chronic model. Further experiments need to be performed to assess the long-term effects of deafening on LGR5 expression and hence translate these findings into the patient’s situation, which are usually chronically injured and establish the best therapeutic window for the treatment. However, based on previous studies showing long term (up to 1 year) survival of SOX2 + SCs in the mouse cochlea, even after injection of high concentrations of kanamycin and furosemide (Oesterle et al., 2008), long term survival of LGR5+ SCs is expected.

### Change in Subcellular Localization of GFP After Deafening: From Nucleus to Cytoplasm

In the present study a change in the intracellular localization of GFP was found in the SCs after deafening. It must be taken into account that Lgr5^GFP^ mice allow for visualization of LGR5+ cells due to a genetic modification that produces EGFP controlled by the promoter of LGR5 (Barker et al., 2007), however, GFP is not fused to LGR5. In the normal cochlea, the GFP expression was mainly localized in the nucleus of SCs. Interestingly, a nuclear localization of GFP is also visible in data of other studies using the Lgr5^GFP^ animal model: in the normal cochlea of Lgr5^GFP^ mice (p0-p19) GFP staining was localized in the nuclei of DC3s (Chai et al., 2011; Shi et al., 2012), and it colocalized with Prox1 (Chai et al., 2011), a transcription factor that is expressed in the nuclei of DCs and pillar cells (PCs) (Bermingham-McDonogh et al., 2006) and with SOX2 (Shi et al., 2012), a transcription factor that controls inner ear development and is expressed in the nuclei of some SCs (Steevens et al., 2019). The observations above further confirm the hypothesis that in both the neonatal and mature organ of Corti in normal-hearing mice, GFP localizes in the nuclei of SCs. After deafening there was a shift of GFP expression to the cytoplasm. The accumulation of GFP or EGFP in the cell nucleus has been previously reported in other models (Seibel et al., 2007) and it is known to occur due to the low molecular weight of the protein which, with only 27 kDa, is able to passively diffuse to the nuclei (Macara, 2001). Nuclear translocation of proteins is a mechanism that occurs in physiological conditions to control gene expression. Many proteins that move to the nuclei have a conserved nuclear localization sequence to signal the translocation through nuclear pore complexes or receptor-mediated import pathways. However, no nuclear localization sequences have been found for GFP or EGFP (Seibel et al., 2007). The change in intracellular localization from nuclei to cytoplasm after deafening could suggest that the nuclear membrane is becoming more permeable, thus promoting leakage of EGFP to the cytoplasm. It could also be due to changes in the concentration of EGFP molecules, which could suggest more EGFP production after deafening.

## Conclusion

In conclusion, this study showed the presence of LGR5+ SCs in the cochlea of adult mice up to p100, which indicates potential endogenous cochlear stem cells with proliferative and regenerative capacities in adulthood. *In vivo* survival of these progenitor cells, after a severe ototoxic event, indicates the availability of target cells for future therapeutic approaches for ototoxic-induced deafness by manipulation of the Wnt- and Notch-signaling pathways. Furthermore, a change in the subcellular localization of GFP after deafening was reported. This report gives further insight into the regeneration potential of the adult deafened cochlea and sets the basis of future therapy to improve hair cell regeneration in hearing-impaired patients.

## Materials and Methods

### Animals

We used 4 C57BL/6 WT mice and 18 heterozygous LGR5-EGFP-CreERT2 (Jackson Laboratory, Stock 008875) mice (Lgr5^GFP^). Nineteen mice were used at postnatal day 30 (p30) and three Lgr5^GFP^ mice at p100. Mice were housed in open cages with food and water *ad libitum* and standard laboratory conditions. All surgical and experimental procedures were approved by the Dutch Central Authority for Scientific Procedures on Animals (CCD:1150020186105).

### Deafening Procedure

Mice were deafened as described previously (Jansen et al., 2013). Non-treated mice were used as normal-hearing controls and consisted of 8 Lgr5^GFP^ and 4 WT p30 mice. The deafened group consisted of 7 Lgr5^GFP^ p30 mice. Normal hearing was confirmed by recordings of ABRs, as described below. Then, 700 mg/kg kanamycin sulfate was injected subcutaneously (stock solution 100 mg/ml in saline). Within 5 min after kanamycin administration, 100 mg/kg furosemide was infused into the tail vein (stock solution 100 mg/ml). Mice were weighed before the deafening procedure and daily after the deafening, since substantial loss of weight can indicate kanamycin-induced kidney failure.

### Auditory Brainstem Responses

ABRs were recorded under general anesthesia using three subcutaneously positioned needle electrodes. The active electrode was placed behind the right pinna, the reference electrode was placed anteriorly on the skull, and the ground electrode was placed in the hind limb. Stimulus generation and data acquisition were controlled by custom-written software involving a personal computer and a Multi-I/O processor (RZ6; Tucker-Davis Technologies, Alachua, FL, United States). Acoustic stimuli consisted of trains of 20-μs clicks with an interstimulus interval of 33 ms. Sounds were presented in an open-field configuration with an electrostatic speaker (TDT ES1) positioned at 3 cm from the pinna. Sound levels were varied from approximately 90 dB peSPL down to below threshold in 10 dB steps. Calibration was performed with Bruel and Kjaer equipment (2203 sound level meter; 1-inch condenser microphone 4132).

### Genotyping

Lgr5^GFP^ transgenic mice were genotyped by isolating DNA from ear tissue. Genomic DNA isolation was performed with DirectPCR lysis reagent (Viagen, Biotech, Los Angeles, CA, United States) according to the manufacturer’s instructions. The primers for PCR amplification were: GFP, forward: CACTGCATTCTAGTTGTGG; and reverse: CGGTGCCCGCAGCGAG. Amplicons were separated by electrophoresis in a 3% agarose gel.

### Cryosectioning and Whole Mount Sample Preparation

Mouse cochleas were harvested after termination by decapitation. Tissues were prepared as described previously (Żak et al., 2016). Briefly, tissues were fixed in 2% paraformaldehyde (Sigma–Aldrich) in phosphate-buffered saline (PBS, pH 7.4) and stored in 2% PFA in PBS at 4°C. Cochleas were decalcified in 270 mM (=10%) EDTA-2Na (Sigma–Aldrich: ED2SS) in dH_2_O at room temperature under constant agitation for 7 days. Cryoprotection of tissues was performed using solutions of increasing concentrations of sucrose (Merck: 1.07653.1000), up to 30%, in PBS (pH 7.4). After subsequent infiltration in a mixture (1:1) of 30% sucrose/OCT compound (Sakura Finetek Europe B.V., Alphen aan den Rijn, The Netherlands) and pure OCT compound, tissues were embedded in OCT and stored at −80°C. Cryosections of 12 μm were cut using a Leica CM3050 cryostat and mounted on microscope slides. For whole-mount samples, tissues were fixed and decalcified as described above. After decalcification, the otic capsule was opened, the lateral wall, Reissner’s membrane, tectorial membrane and modiolus were removed and the basilar membrane containing the organ of Corti was dissected into individual half-turns.

### Immunofluorescence Microscopy

Immunofluorescence staining was performed on cryosections and whole-mount dissections. The tissues and slides were washed with blocking solution (2% donkey serum and 0.1% triton X-100 in PBS). Specimens were incubated with primary antibodies, anti-myosin VIIA (MYO7A, 1/300, rabbit, Proteus Biosciences, 25-6790) and anti-GFP (1/200, goat, Abcam, ab5450) overnight at 4°C. Later, slides and tissues were washed with blocking solution and incubated with secondary antibodies donkey-anti Rabbit-Alexa 594 (1/500, Invitrogen, A-21207), donkey-anti Goat-Alexa 488 (1/200, Abcam, AB150129), and DAPI solution (1/500, Abcam, AB228549) for 90 min at room temperature. Lastly, specimens were washed in PBS and mounted in Vectashield Antifade Mounting Medium (Vector laboratories, H-1000). Slides were imaged using a Zeiss LSM700 Scanning Confocal Microscope. Apical, middle, and basal regions were calculated by measuring the total length of each cochlear duct in the whole-mount dissections and calculating 25% (apex), 50% (middle), and 75% (basal) distance from the apical end. Three-dimensional image reconstruction of Z-stacks and PCC analyses of DAPI and GFP signals were performed using ImageJ software.

### Cell Counting

Cells were counted by three independent raters using whole-mount dissection immunofluorescence staining images. The total number of IHCs and OHCs were counted by analyzing MYO7A+ cells. SCs were counted by assessing LGR5+ cells located in the DC3s. Cells were counted in each of three cochlear segments (apical, middle and basal). Density (cells per 100 μm) was then calculated for each segment and numbers were normalized vs. normal-hearing littermates and shown in percentages.

### Statistical Analysis

Significance of differences in cochlear tissues between the deafened and normal-hearing mice was tested by repeated measures ANOVA with ototoxic treatment as between-group factor and cochlear location (basal, middle, apical) as within-animal factor. These analyses were performed in SPSS statistics version 27 for windows (IBM Corp., Armonk, NY, United States). Results were considered statistically different when the *p*-value <0.05.

## Data Availability Statement

The raw data supporting the conclusions of this article will be made available by the authors, without undue reservation.

## Ethics Statement

The animal study was reviewed and approved by the Utrecht Animal Welfare Body of Utrecht University and UMC Utrecht.

## Author Contributions

NS-C, RY, and LS: conceptualization. RY, FH, and HV: methodology. NS-C, RY, FH, ES, DR, and LS: investigation. NS-C and HV: formal analysis. HV, RS, and LS: resources, project administration, and funding. NS-C, HV, and LS: writing—original draft. HV and LS: writing—revision and editing and project supervision. All authors contributed to the article and approved the submitted version.

## Conflict of Interest

The authors declare that the research was conducted in the absence of any commercial or financial relationships that could be construed as a potential conflict of interest.

## Publisher’s Note

All claims expressed in this article are solely those of the authors and do not necessarily represent those of their affiliated organizations, or those of the publisher, the editors and the reviewers. Any product that may be evaluated in this article, or claim that may be made by its manufacturer, is not guaranteed or endorsed by the publisher.
